# Comparative genomics of closely related *Salmonella enterica* serovar Typhi strains reveals genome dynamics and the acquisition of novel pathogenic elements

**DOI:** 10.1186/1471-2164-15-1007

**Published:** 2014-11-20

**Authors:** Kien-Pong Yap, Han Ming Gan, Cindy Shuan Ju Teh, Lay Ching Chai, Kwai Lin Thong

**Affiliations:** Institute of Biological Sciences, Faculty of Science, University of Malaya, 50603 Kuala Lumpur, Malaysia; Laboratory of Biomedical Science and Molecular Microbiology, Institute of Graduate Studies, University of Malaya, 50603 Kuala Lumpur, Malaysia; Department of Medical Microbiology, Faculty of Medicine, University of Malaya, 50603 Kuala Lumpur, Malaysia; School of Science, Monash University Malaysia, Jalan Lagoon Selatan, Bandar Sunway, 46100 Selangor Malaysia

**Keywords:** *S*. Typhi, Typhoid, Genomes, Comparative genomics, Pathogen, *zot*, SPI, T6SS, Phylogenetic, *Enterobacteriaceae*, Sequence, Evolution, Protein, Strain, Variation, Virulence, Infection, Protein modelling

## Abstract

**Background:**

Typhoid fever is an infectious disease of global importance that is caused by *Salmonella enterica* subsp. *enterica* serovar Typhi (*S*. Typhi). This disease causes an estimated 200,000 deaths per year and remains a serious global health threat. *S*. Typhi is strictly a human pathogen, and some recovered individuals become long-term carriers who continue to shed the bacteria in their faeces, thus becoming main reservoirs of infection.

**Results:**

A comparative genomics analysis combined with a phylogenomic analysis revealed that the strains from the outbreak and carrier were closely related with microvariations and possibly derived from a common ancestor. Additionally, the comparative genomics analysis with all of the other completely sequenced *S*. Typhi genomes revealed that strains BL196 and CR0044 exhibit unusual genomic variations despite *S*. Typhi being generally regarded as highly clonal. The two genomes shared distinct chromosomal architectures and uncommon genome features; notably, the presence of a ~10 kb novel genomic island containing uncharacterised virulence-related genes, and *zot* in particular. Variations were also detected in the T6SS system and genes that were related to SPI-10, insertion sequences, CRISPRs and nsSNPs among the studied genomes. Interestingly, the carrier strain CR0044 harboured far more genetic polymorphisms (83% mutant nsSNPs) compared with the closely related BL196 outbreak strain. Notably, the two highly related virulence-determinant genes, *rpoS* and *tviE*, were mutated in strains BL196 and CR0044, respectively, which revealed that the mutation in *rpoS* is stabilising, while that in *tviE* is destabilising. These microvariations provide novel insight into the optimisation of genes by the pathogens. However, the sporadic strain was found to be far more conserved compared with the others.

**Conclusions:**

The uncommon genomic variations in the two closely related BL196 and CR0044 strains suggests that *S*. Typhi is more diverse than previously thought. Our study has demonstrated that the pathogen is continually acquiring new genes through horizontal gene transfer in the process of host adaptation, providing novel insight into its unusual genomic dynamics. The understanding of these strains and virulence factors, and particularly the strain that is associated with the large outbreak and the less studied asymptomatic Typhi carrier in the population, will have important impact on disease control.

**Electronic supplementary material:**

The online version of this article (doi:10.1186/1471-2164-15-1007) contains supplementary material, which is available to authorized users.

## Background

Typhoid fever is a human systemic infection that is caused by *Salmonella enterica* subsp. *enterica* serovar Typhi (*S*. Typhi). This human-restricted and highly adapted pathogen is transmitted via the oral-faecal route. *S*. Typhi is responsible for 21.7 million infections and results in approximately 217,000 deaths worldwide annually [1]. The disease primarily causes acute systemic infection with life-threatening complications, and the recovering patient may develop into a chronic carrier state [2].

Typhoid is endemic, with periodic outbreaks and sporadic cases occurring in developing countries, particularly in southeast Asia, south central Asia, Latin America and southern Africa where sanitary conditions are poor [1]. Among the 13 states of Malaysia, Kelantan has a significantly higher incidence of typhoid fever [3]. A large typhoid outbreak occurred in Kelantan state, which resulted in 735 cases of infection and two deaths in a short period of 3 months (from April to June of 2005) [3]. Previously, pulsed-field gel electrophoresis (PFGE) revealed close genetic relatedness between a *S*. Typhi strain that was isolated from an asymptomatic carrier in 2007 and a strain that originated from a patient during the 2005 outbreak in Kelantan (unpublished data).

Human carriers are the main reservoirs of *S*. Typhi transmission, but the genetic basis, and the underlying mechanisms in particular, are unclear [4]. It has been suggested that a carrier strain will likely lack gene acquisition capabilities and have little fitness advantages compared with those strains causing symptomatic infections because the human reservoir is small and physiologically isolated [4, 5]. Therefore, it is of great interest to know as to what extent these closely related strains differed or shared in its genomic contents despite being isolated from these two distinct epidemiological settings. In 2008, a *S*. Typhi strain was isolated from a sporadic case in Kuala Lumpur. This genome has been sequenced earlier [6], and PFGE analyses showed that this strain is more distantly related to the outbreak and carrier strains, but the epidemiological link is unknown (unpublished data).

Recent whole-genome sequencing of *S*. Typhi has demonstrated that the pathogen shows limited genetic variation with little evidence of purifying selection, antigenic variation or recombination between isolates [4, 7]. This clonal pathogen, however, is associated with varying degrees of disease severity in different regions [8]. Previous PFGE studies have also demonstrated genome size variations and distinct PFGE patterns in relation with fatal and non-fatal typhoid cases [9, 10]. Although the health conditions of the host cannot be completely ruled out, various reports have suggested that the gain and loss of genes through mutations and gene transfers that have occurred independently in different lineages have markedly contributed to the varying pathogenic potentials [4, 11]. However, these important factors are poorly understood because there is limited genomic information for *S*. Typhi, particularly involving strains that are associated with diverse epidemiological settings. Its genomic heterogeneity is likely due to the adaptation of the pathogen to the host and its exposure to mobile elements, such as bacteriophages [12]. Organisms having common core genomes could differ in their dispensable (strain-specific) genes, reflecting their unique physiological and virulence properties [13, 14]. Although not all genetic variations are essential for adaptation, some dispensable genes are believed to be responsible for conferring fitness advantages to the pathogen to thrive in its host. Horizontal gene transfer is also thought to be the predominant force in bacterial evolution, which contributes to novel gene acquisition. The acquired genes provide new characteristics, which either aid in host adaptation and persistence or enhance virulence capabilities [12–15]. A previous study on the pan-genome of *Salmonella enterica* revealed that the pan-genome (total known genomic content) of all strains will continue to increase as new genomes are sequenced [16]. With the availability of robust next-generation sequencing technologies, high quality whole genome sequences can be generated and analysed, which will be especially useful for capturing fine variations among highly conserved *S*. Typhi strains. Multiple whole genome sequence comparisons of closely related strains will not only lead to the better understanding of their relationships but also provide novel insights into the functional roles of strain-specific genes.

In this study, we performed detailed and comprehensive comparative functional analyses of three previously sequenced genomes of *S*. Typhi strains that were isolated from typhoid patients during a large outbreak in 2005, a sporadic case in 2008 and an asymptomatic carrier from Malaysia, where typhoid is endemic. These Malaysian *S*. Typhi strains were compared with previously published *S*. Typhi genomes with the following aims: 1) to determine and describe the genomes signatures and conserved and unique regions of the strains that were being studied; 2) to elucidate the phylogeny and genetic relatedness of these Malaysian strains compared with 17 other published strains using phylogenomic analysis; 3) to compare those strains that have been associated with various epidemiological settings (outbreak, carrier and sporadic cases), and particularly the regions of plasticity that may contribute to the varying pathogenic potentials; 4) to identify potential novel pathogenic factors that are harboured by the analysed strains; 5) to provide insight into the possible differential functionalities of the genes, focusing mainly on virulence- and persistence-related genes based on non-synonymous SNPs of closely related strains and particularly on carrier strains to gain insight into the persistence of the carrier state; and 6) to understand how the potential nsSNPs affect protein structures and functions. The data that are generated will be useful for the profiling of strains, marker development and the increased understanding of outbreak, sporadic and less studied asymptomatic typhoid carriage infection.

## Results and discussion

### General genome signatures of *S*. Typhi in association with outbreak, sporadic case and carrier

The genomes of the three previously sequenced Malaysian *S*. Typhi strains from different sources were compared to identify potential genomic features that may help to elucidate the different disease outcomes. One strain was isolated during the largest outbreak in the country, one from a carrier (food handler) during typhoid surveillance following the outbreak, representing the carrier state, and one from a sporadic case in the metropolitan area of Kuala Lumpur, which has a relatively lower incidence of typhoid. However, because of the limited numbers of strains studied and absence of information regarding the pan-genome of the *S*.Typhi population, the genetic differences observed should be taken with caution. The aim of the detailed comparative analysis was to provide a better understanding and insights into the unusual genome dynamics of *S*. Typhi, a highly clonal organism.

**Figure 1:**
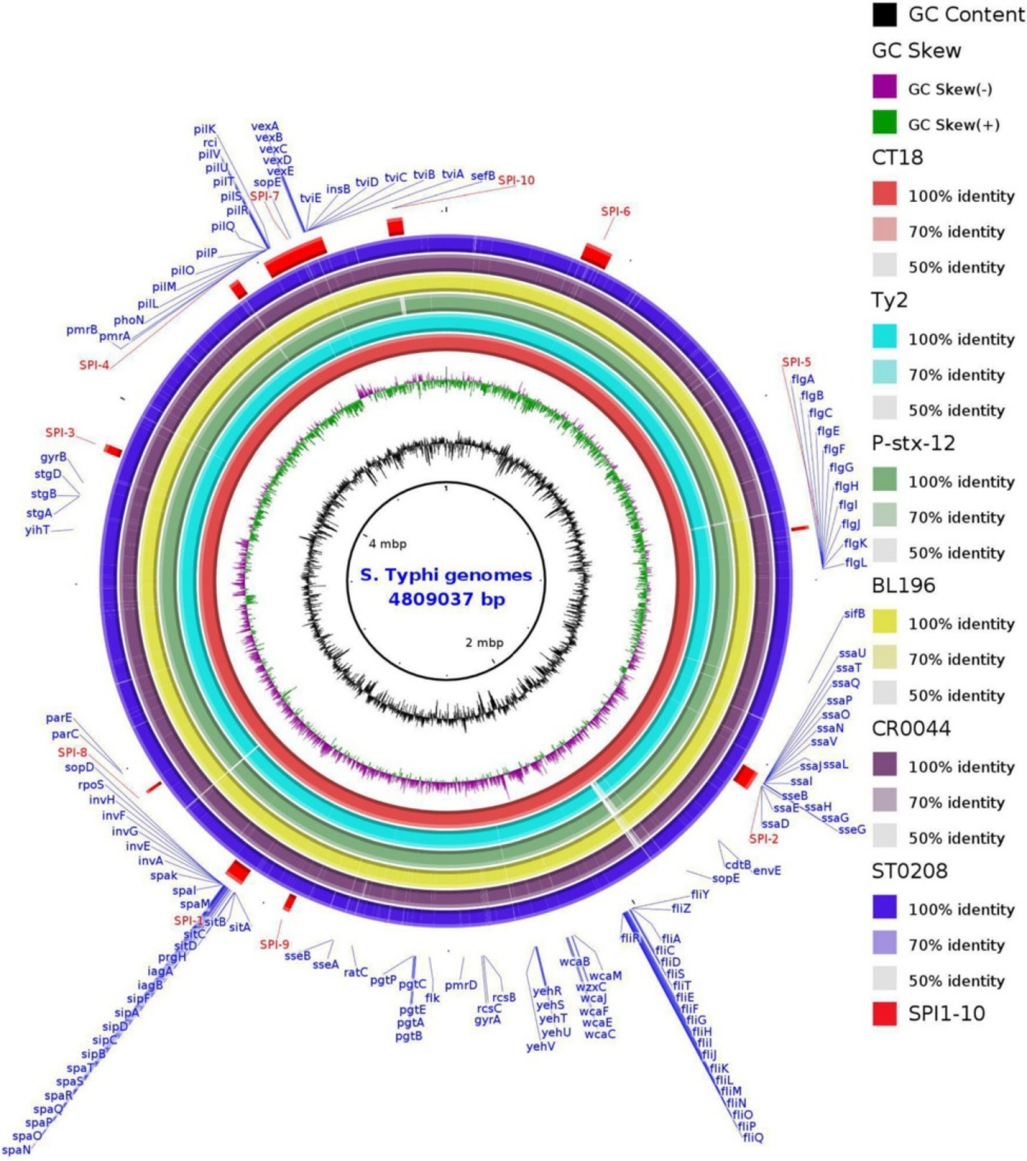
**Circular genomes representation map and genome comparison of** ***S*** **. Typhi (CT18, Ty2, P-stx-12, BL196, CR0044, ST0208).** The circle is divided into arcs representing the sequence of the chromosomes of all six genomes as labeled. The inner ring shows the coordinates in scale and total genomes size of the reference sequence, CT18 in scale (mbp), with black histogram bar representing GC content and purple-green histogram bar representing GC deviation. Orthologs for each genomes with respect to CT18 (innermost red arch) are showed in order (inside-out) with the percentage of similarity based on nucleotide sequences (100%, 70% and 50%, colour tone from darkest to lightest) as indicated on the legend located at the right of the figure. The outermost arch in red represents the location of SPI1 to SPI10 in relative to CT18 and labeled in red. The position of the genes related to pathogenicity and host persistent are shown and labeled in blue at the edge of the rings.

Previous genome sequencing analyses have generated high-quality assemblies with an average genome coverage of 100× for the 3 Malaysian *S*. Typhi genomes, including BL196 (an outbreak strain that was isolated from a blood sample; Genbank accession number AJGK00000000.1) [17], CR0044 (a strain that was isolated from a stool sample of a carrier; Genbank accession number AKZO00000000.1) [18] and ST0208 (a sporadic strain that was isolated from a stool sample of a typhoid case; Genbank accession number AJXA00000000.1) [6]. The approximate predicted genome sizes and average guanine-plus-cytosine (G + C) contents of all 3 genomes ranged from 4.7 Mb to 4.8 Mb and 52.0% to 53.2%, respectively (Additional file 1). These genomes form a single and circular chromosome with no plasmids detected. An *in silico* multi-locus sequence typing (MLST) analysis classified both BL196 and CR0044 as ST 1 and ST0208 as ST 2, which are the main sequence types that have been associated with the worldwide distribution of *S*. Typhi out of the 4 STs that have been identified to date [19]. The predicted coding sequences (CDSs) of the genomes based on RAST subsystem-based annotations varied from 4,875 to 4,890 with an average coding percentage of 86.0%. The average sizes of the CDSs were similar (ranging from 810 bp to 875 bp), indicating that the size differences among the genomes are largely attributable to a number of CDSs and intergenic regions. Approximately 12% of the CDSs were annotated as uncharacterised proteins (Additional file 1). Some of these genes (4.2%) were observed to vary from one strain to another. These “dispensable” genomes carried genes that were present in one or more strains and could even be unique to a single strain [20], indicating a possible open pan-genome of *S*. Typhi. In general, the chromosomes of the three assembled genomes exhibited overall structural conservation and colinearity with each other as evidenced by the homologous and conserved regions that were shared and the very small strain-specific regions (Figure 1), which may harbour genes that are relevant to the specific adaptations and fitness advantages of each of the strains. These regions most likely represent DNA that was acquired during events of HGT that may provide the strains with greater metabolic versatility or even virulence capabilities, as will be further discussed in the plasticity section.

### Comparative genomics of *S*. Typhi

Genomic comparisons were performed on the three Malaysian *S*. Typhi strains and the three completed *S*. Typhi genomes (the only full genomes available at the NCBI database to date), CT18 (Genbank accession number AL513382) [19], Ty2 (Genbank accession number AE014613) [21] and P-stx-12 (Genbank accession number CP003278) [22]. The comparisons of the studied strains with the reference genomes allowed for the elucidation of novel and additional genes that are carried by the Malaysian *S*. Typhi genomes that may be of significance. We have analysed the shared and unique genes of all six genomes to determine their distinct virulence and pathogenic features. As expected, the six genomes exhibited high similarities and syntenies with each other with limited evidence of genomic rearrangements, which collectively indicate stable genomic structures. The majority of the ORFs from the compared genomes were part of a conserved genomic core, in which 4532 ORFs were shared among all of the genomes. These shared ORFs provide clear evidence of conservation among the genomes of the *S*. Typhi strains. The rest of the unshared ORFs or accessory genes are present in one or more strains, which represent the salient differences in the genomes. Most of these ORFs (4.2% to 4.9%) that were harboured by each genome were annotated as hypothetical proteins (50% to 75%). Our extended homology analysis has shown that the remaining unshared ORFs (25% to 50%) were likely to encode for functional proteins from diverse categories, including virulence-related proteins, secretory proteins, conserved domain proteins, transporter proteins and phage proteins among others. These considerable portions of the genomes could provide important functional clues for understanding the virulence and persistence of the pathogen more clearly, anticipating the need for extensive future studies focusing on their possible roles in bacterial pathogenesis. However, the numbers of shared and unshared ORFs may have been underestimated because the genomes were incomplete. Among the shared genes that were found between strains BL196 and CR0044, uncommon ORFs that encoded for the VI Icm-F secretion protein, Icm-F-related protein and type VI secretion protein EvpB were identified whose products are related to the type VI secretion system. The genes shared 99% similarity with the type VI secretion protein of *Salmonella* Typhimurium strain D23580 [23] and were only found in BL196 and CR0044. This protein was recently recognised as one of the main virulence determinants in *Burkholderia pseudomallei*, *Legionella pneumophila* and *Vibrio cholerae,* but its function in *S*. Typhi remains to be elucidated [24, 25]. T6SS genes are believed to be involved in either structural components of the secretory apparatus, secretory products or assisting with protein translocation; for example, providing the energy to push substrates through the channel of the apparatus [26]. These genes are also proposed to be involved in surface reorganisation, enhancing adherence to epithelial cells, intracellular multiplication and human macrophage killing [26–28]. Other T6SS clusters were found intact as in reference genomes. The high similarities of the genetic contents of BL196 and CR0044 with minor variations, and particularly the presence of unique accessory genes (in addition to SNPs, which are discussed in another section), are in agreement with the PFGE pulsotype data, which revealed that both strains are genetically similar, showing a difference of only one band (Additional file 2).

The chromosome of *Salmonella enterica* is commonly integrated with a large portion of horizontally acquired DNA apart from its core, which are termed the Salmonella pathogenicity islands (SPIs) [29]. These acquired SPIs have led to divergence and host restriction similar to those in *S*. Typhi. The identification of conserved SPIs and their variations have important implications in a wide range of microbiological applications, such as antigen and marker discovery and the identification of essential genes and their respective traits. In this study, we have annotated all SPIs (SPI-1 to SPI-10) and its genetic variant of *S*. Typhi in the genome, which is characterised by its deviated GC content, flanking by tRNA genes and the presence of phages, integrases, recombinases and genes that are related to DNA integration. Although all the SPIs [1–10] (Figure 1) were detected in the genomes, there were marked variations. The presence of a large number of transposition-related genes in these SPIs suggests that the sites may be actively involved in the integration and transposition of genetic elements, which drive genetic variation. Interestingly, our comparative analysis revealed that the carrier strain P-stx-12 lacks a ~10 kb *prpZ* cluster and adjacent gene clusters harbouring 14 ORF with a deviated GC skew of 49.2 % at SPI-10 but remains fully intact in our carrier strain (Figure 1). Previously, a *prpZ* cluster deletion study showed that the mutant has a significantly lower survival rate compared with the parental strain, which may be due to a signalling pathway that controls the long-term survival of *S*. Typhi in host cells, and particularly, the survival in human macrophages [30]. In fact, our results support this study with the fine-tuned postulation that the deletion led to reduced virulence that enabled the carrier strain to coexist with the host; for example, in the tissue of gall bladder. This possibly explains why long-term survival in the macrophage is no longer necessary, which is presumably because the pathogens have colonised and persisted in other cells of the host during adaptation. However, the deletion was not detected in our carrier strain, suggesting that the genes may not be the only factors that are relevant to a carrier state. Furthermore, the region is flanked by multiple transposases, integrases, ligases and uncharacterised proteins, which are known to be involved in transposition. Additionally, genes coding for the DNA mismatch repair protein mutC and transposase were identified both upstream and downstream of the cluster, suggesting that the region could have possibly been acquired earlier during horizontal gene transfer. The presence of a DNA mismatch protein gene has been previously implicated to be involved in modulating recombination events by incorporating or inhibiting the transfer of mobile genetic elements [31]. The deletion of the gene cluster and the presence of a large number of genes that are related to transposition indicate that SPI-10 may be unstable and prone to excision similar to the precise excision of the crucial SPI-7, which has been recently reported in *S*. Typhi [32], suggesting that gene deletion may be important to the host adaptation of this organism, although independent acquisition or gene gain by other strains cannot be completely ruled out. These findings expand upon previous studies, which have reported that other SPIs are relatively stable in the genome [4], highlighting the importance of a future evaluation of the stability of the other SPIs. Apart from these remarkable differences, the comparison of the two carrier strains, CR0044 and P-stx-12, revealed that CR0044 carries several additional genes that were not identified in P-stx-12 that encode for unknown functions and phages that are present in the phage region. Almost all of the potential major virulence and persistence-associated genes have homologues in P-stx-12, suggesting that they are not specifically associated with the unique persistence of the carrier strains but are common to *S*. Typhi. The genomic structure of the sporadic strain ST0208 is more conserved in comparison and has relatively fewer dispensable ORFs, which are mainly genes that code for hypothetical proteins and phages, indicating the conservation of large numbers of genes, which is essential for strict host adaptation and virulence optimisation.

### Phylogenomics of *S*. Typhi revealed shared common ancestry

**Figure 2:**
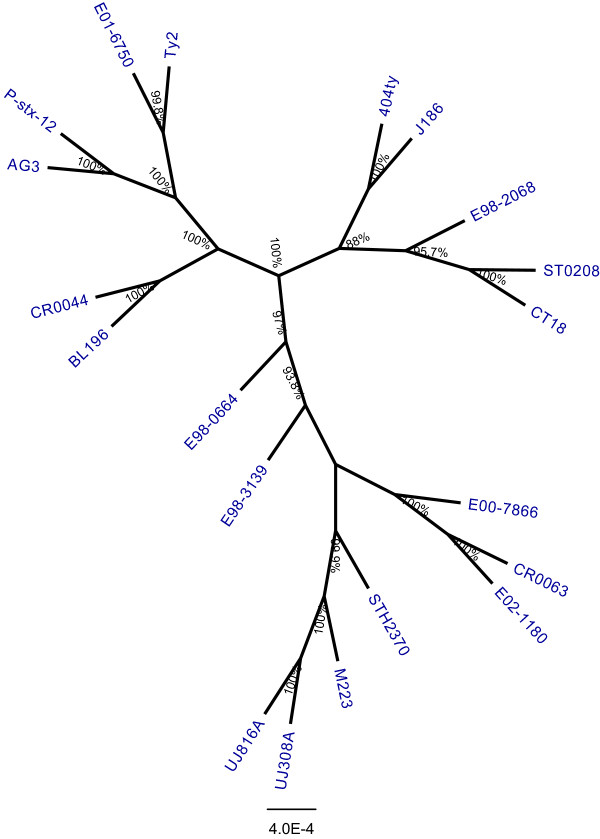
**Phylogenomic tree inferred by approximately-maximum-likelihood method from the aligned core genomes.** Multiple genomes alignments were generated by mapping genome sequences of the 20 global *S*. Typhi strains against CT18 at all sites relevant for phylogenomic analysis using RealPhy [78]. Phylogenetic tree (unrooted) was inferred via approximately-maximum-likelihood method using FastTreeMP [79]. Strains studied were labelled in blue. Bootstrap support values shown at each node.

We determined a core genome-based phylogeny by mapping 20 query genomic sequences against CT18 into a single non-redundant alignment of 3,495,681 bp (Figure 2) (see Methods). The phylogenomic tree showed that the outbreak strain BL196 and carrier strain CR0044 were closely related and could be differentiated by only 50 SNPs. These data are in agreement with our PFGE results that showed that both strains are highly related (Additional file 2). The observed close phylogenetic relationship between these two strains is consistent with our earlier speculation that the large outbreak that occurred in 2005 shared a common ancestor strain with the typhoid carrier that may have been circulating for a long period in the country. It is challenging to determine how these two strains are related, considering their short-term evolutionary relationship. However, three evolutionary postulations are possible. First, the carrier strain may have been derived from the outbreak strain. Second, the carrier strain may have long existed in a carrier who served as a reservoir and the source of the outbreak. Finally, both of these strains may have diverged independently from a common ancestor, which was possibly harboured in a long existed carrier to give rise to two independent cases, considering the geographical proximities. Notably, these two highly related strains with unique gene repertoires clustered with 4 epidemiologically and geographically unrelated strains from India (P-stx-12), Russia (TY2), Vietnam (AG3) and Senegal in west Africa (E01-6750) (Figure 2). Interestingly, all of these strains, including BL196, CR0044, P-stx-12, Ty2 and E01-6750, were subtyped as ST 1 (we could not establish the ST from the genomic sequence of AG3). Conversely, the Malaysian sporadic *S*. Typhi strain ST0208 clustered closely with the multidrug-resistant strain CT18 from Vietnam together with other geographically related strains from Indonesia (404ty and J185) and Bangladesh (E98-2068) (Figure 2), which were all subtyped as ST 2, suggesting the possible movement of clonally related strains among the southeast Asian countries. Such close genetic relatedness that is based on macrorestriction and SNP typing analyses has been previously reported [33, 34]. The rest of the genomes were clustered as ST 2. From the analysis, we found no temporal or geographical signals, but the sequence types was highly correlated with the phylogenomic clustering. Apparently, the cluster subtyped as ST 2 could be further differentiated into two clusters according to the phylogenomic analysis but was limited in the current MLST scheme for *S*. Typhi. These results indicate that the phylogenomic analysis has much better resolution power compared with MLST in separating highly clonal strains in *S*. Typhi. This information is essential for devising a better set of MLST alleles for improved molecular typing. These data further support the widespread distribution of ST 1 and ST 2 as the major genotypes that occur worldwide, although rarely, ST 3 and ST 8 have also been isolated in a previous study [19]. It is important to note that the two carriers, CR0044 and P-stx-12, belonged to two different subtypes, reflect the non-universality of the genotypes relevant for carrier state transformation.

### Genome plasticity (insertion sequences, phages and CRISPRs)

Our analyses of the relationships among the strains and their genomic variations are further supported and extended to the study of the genome plasticity of *S*. Typhi. In many organisms, genomic plasticity is commonly observed, but *S*. Typhi has generally demonstrated few variations compared with other *Salmonella* spp. [35]. However, in our study, we detected considerable genetic variations, predominantly involving IS elements, phages and CRISPRs. Marked variations in the numbers and types of IS elements were detected. *IS*200 (200, 200 F, 200C, 200G and 200H) and *IS*1541 (1541A, 1541B, 1541C and 1541D) were both abundant in the three Malaysian *S*. Typhi genomes. The IS elements, such as *IS*200, have been widely used as molecular markers for subtyping due to their genome-wide distributions and high levels of diversity, but their roles in modulating the gene expression in *S*. Typhi have yet to be clarified [36]. Recent studies on enterohaemorrhagic *Escherichia coli* O157 have shown that the presence of IS could play a role in the gene inactivation and immobilisation of incoming phages and plasmids, leading to the diversification and evolution of the bacterial genome [37]. IS elements have also been shown to affect the expression of neighbouring genes and induce genomic rearrangements (deletions, inversion and duplications) [38]. However, little is understood about their roles in modulating virulence and gene expression in *S*. Typhi. The variations that were detected may provide clues on how these differences affect the virulence and fitness strategies of the pathogens.

The *S*. Typhi strains BL196, CR0044 and ST0208 carry eight, seven and eight phages, respectively. We have identified substantial phage variations among the *S*. Typhi genomes. One of the differentiating features was a distinct set of prophages that were harboured by both BL196 and CR0044, which rendered them less unique compared with ST0208 with the exception of a few ORFs that encoded for phages and hypothetical proteins. Interestingly, the phages that were carried by ST0208 had relatively shorter in lengths (in bp) compared with the phages that were identified in the other strains. As expected, both of the closely related strains (BL196 and CR0044) had highly similar phage contents and carried an additional intact *Salmonella* phage RE-2010 with uncommon ORFs, which mainly contained genes coding for hypothetical proteins, phage proteins, prophage-like proteins, repressor proteins, excisionases, terminases and integrases. The variations that were detected in the numbers of predicted prophages and prophage-like regions illustrated the dynamics of phage gain and loss that distinguished one strain from the others, indicating that phages may play important roles in genomic diversity. By correlating phylogenomic analysis and whole genome sequence alignments, this region appears to show the typical gain and loss of sequences during the course of genomic evolution. This evolutionary relationship is consistent with the phage variations, providing a useful framework for investigating the relationships of strains and their respective phenotypes. The phage was likely acquired prior to the divergence of the common ancestor of both BL196 and CR0044 (Figure 2) through horizontal gene transfer rather than phage loss. Alternatively, due to the advantageous roles of the HGT events, the phage proteins that were acquired by the strains could promote their *in vivo* survival and pathogenesis [39]. Among the detected phages, the two typical *S*. Typhi phages, Gifsy-2 and Fels-2, were both found to be intact and conserved in all six of the *S*. Typhi genomes, suggesting that these regions may play essential roles and provide fitness advantages to the pathogen. Apart from the intact phages, 3 incomplete phages, including Burkholderia_phage_BcepMu, Enterobacteria_phage_cdtI and Lactococcus_phage_bIL312, were also observed in the six genomes, suggesting that these common phages were likely present in their common ancestors and may be relevant for host adaptation and survival.

Clustered regularly interspaced short palindromic repeats (CRISPRs together with the *cas* genes) were recently found to be important in bacteria as a primary defence strategy against foreign nucleic acids, including phages and conjugative plasmids [40]. In fact, CRISPR regions have been found to be integrated in response to infecting phages. These regions are known to have hypervariable genetic loci due to the high diversities of the interspaced regions between the palindromic repeats and frequently match to phage and other extrachromosomal elements. The presence of CRISPRs in genomes may affect short-term phenotypic changes and mediate long-term sublineage divergences [40]. CRISPR regions were identified in all three of the Malaysian *S*. Typhi genomes using CRISPRs Finder online (crispr.u-psud.fr/Server/). The identified regions were found to be located around the CRISPR-associated protein *cas1* and flanked by genes coding for alkaline phosphatase isoenzyme conversion aminopeptidase and clusters of CRISPR-associated genes, including *cas* and *cse*. CRISPR_1 was identified with the palindromic repeats, showing strikingly high similarities among all of the analysed *S*. Typhi genomes with minor variations in spacers, indicating the important evolutionary conservation of the *S*. Typhi strains. Apparently, the gene orders of CRISPRs are also conserved among the genomes. The sporadic *S*. Typhi strain ST0208 harbours a shorter CRISPR region (designated as confirmed CRISPRs by CRISPRs Finder) of 333 bp in length compared with CT18 (385 bp), Ty2 (394 bp) and P-stx-12 (394 bp). The CRISPR region of ST0208 has identical palindromic repeats and a repeat length compared with the other genomes but lacks one spacer. Similar observations were also observed in CT18, which had shorter spacers compared with the rest, which is in agreement with phylogenomic analysis, that CT18 is genetically related to ST0208 and cluster together with Ty2 and P-stx-12, which were found to possess CRISPR regions of identical lengths. A previous study showed that the addition or deletion of spacers is able to modify the phenotype of phage resistance [41]. Alternatively, non-identity spacers have been suggested to mediate the interactions between CRISPRs and phages [42]. The diversity of spacers in CRISPRs of *S*. Typhi may be relevant to other interesting roles that are yet to be understood. Interestingly, additional CRISPR-like regions (designated as possible CRISPRs by CRISPRs Finder) were observed to be identical in the two closely related strains, BL196 and CR0044. We found that all of the *S*. Typhi spacers that were analysed showed homology to many eukaryotic sequences, extrachromosomal sequences and phages, supporting the immunity roles of CRISPRs against phages and other incoming DNA. Variations in CRISPR regions were identified, including those in BL196 and CR0044, providing evidence of evolutionary relevance that is useful for distinguishing between closely related strains and inferring ancestral relationships.

### Putative pathogenomic island harbouring *zot*, a potential novel virulence-determining factor

**Figure 3:**
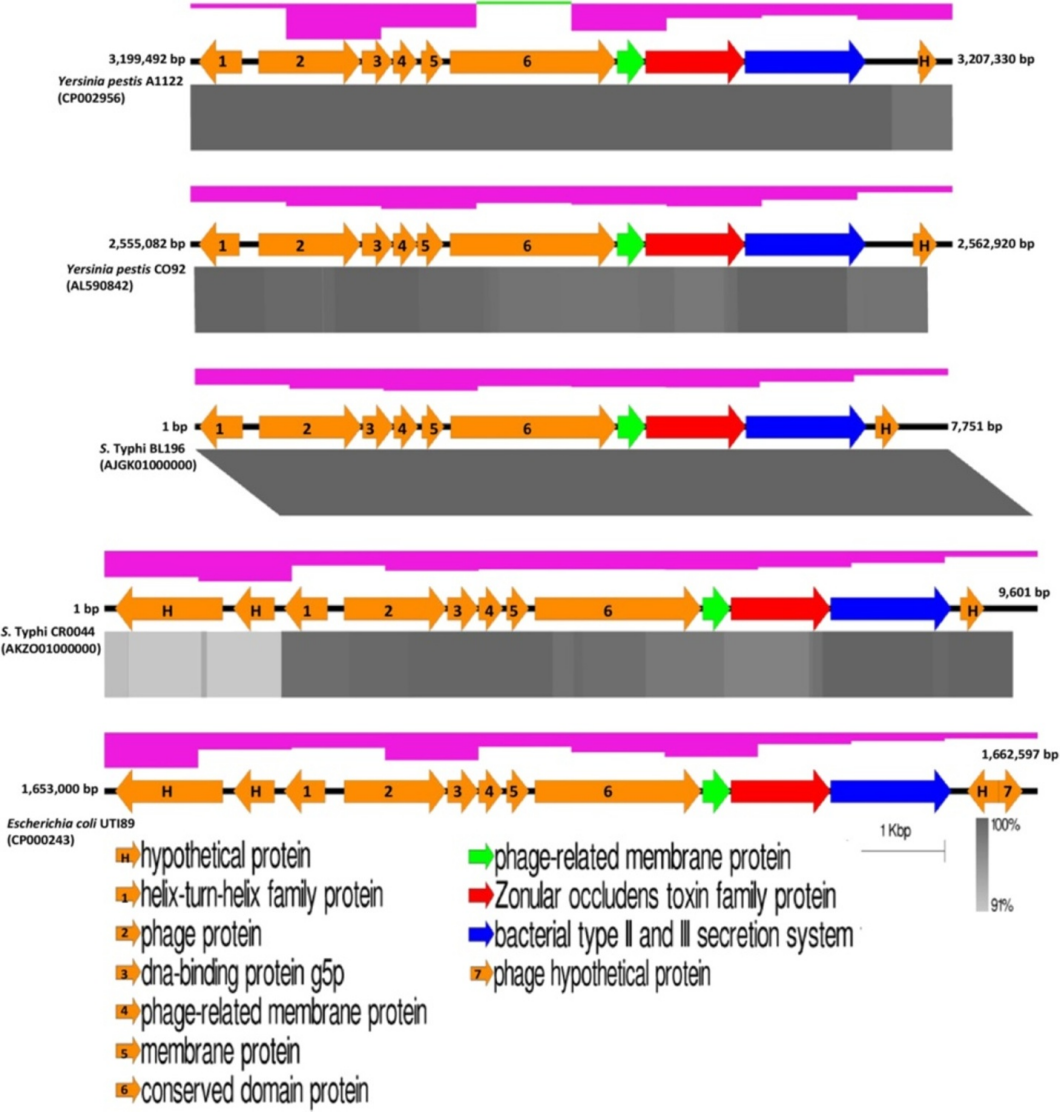
**Schematic representative novel putative pathogenomic island harboring** ***zot*** **of CR0044 and BL196.** 7.7-kb and 9.6-kb genomic island fragment of strain BL196 and CR0044, respectively. The schematic diagram shows the presence of various virulence associated genes detected, particularly *zot* and tBLASTx comparison (represented by the grey bars with varying colour intensity) with *Yersinia pestis* A1122 and *Yersinia pestis* CO92. The position of the regions in the genomes is labeled (bp). The arrow bars denode annotated genes as in the legend based on BLAST classification (the BLASTP analysis was carried out across a non-redundant protein database in GenBank). The arrow direction showed transcription direction of the gene. The green and pink blocks above the arrow bars denode GC deviation.

A total of eight (7.7 kb; GC 42.3%) and 10 ORFs (9.6 kb; GC 40.5%) were identified in BL196 and CR0044, respectively, with eight commonly shared (100% sequence similarities) ORFs being identified (Figure 3) compared with the other genomes. These clusters were predicted to be Genomic Islands (GIs) using IslandViewer (predicted by at least one method) [43]. GIs are non-self-mobilising elements that code for proteins with diverse functions that may be integrated or excised, thus playing important roles in bacterial diversification and adaptation. The predicted features of the identified GIs resemble those of the previously reported pathogenicity islands, such as the presence of ISs, integrases, transposases and deviated GC contents [44]. The GIs were found to harbour a novel gene coding for the zonular occluden toxin family protein (*zot*), that is convergently oriented with respect to its flanking genes coding for a conserved domain protein and bacterial Type II and III secretion proteins respectively, suggesting their involvement in the transportation of Zot toxin into the host intestine. Intriguingly, the genomic elements together with the hypothetical proteins shared remarkable sequence similarities of >90% with *Yersinia pestis* A1122 and *Yersinia pestis* CO92 [45, 46], the causative agents of the Black Death (Figure 3). We further screened for the prevalence of this gene in 41 *S*. Typhi strains from diverse locations that were collected over a span of 25 years from 1983–2008 and remarkably, *zot* is only present in strains BL196 and CR0044 (Additional files 3, 4 and 5). This is in agreement with our speculations that both the clonally related strains are responsible for the outbreak and carrier cases. Future studies could be carried out to fully characterise the GIs and their relevant roles in the pathogenesis of *S*. Typhi.

**Figure 4:**
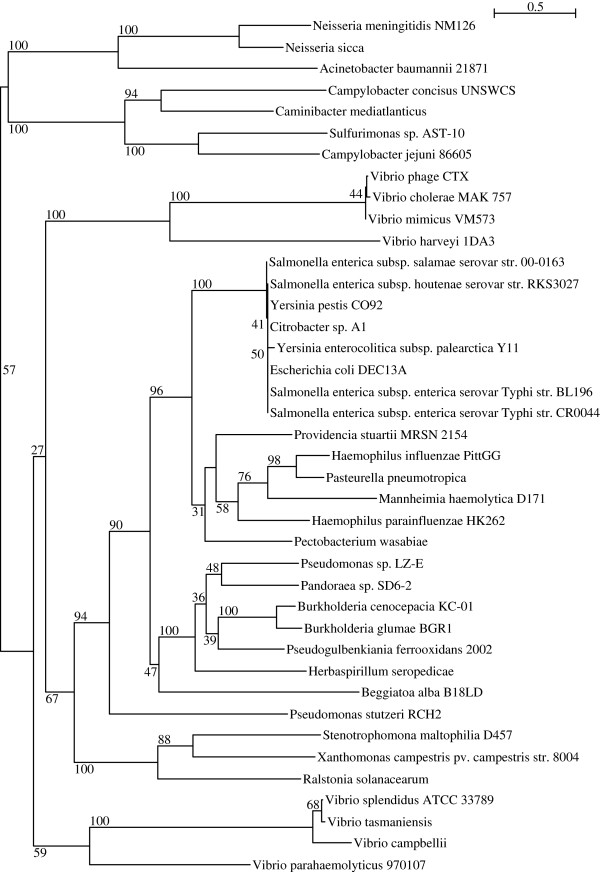
**Phylogenetic tree of** ***zot*** **.** Phylogenetic analysis of *S*. Typhi *zot* with *zot* genes of 40 closely and distantly related bacteria strains using the Approximate-Maximum-Likelihood method. Branch support was assessed with 1000 bootstrap replicates. Bootstrap support values shown at each node.

*Salmonella* spp. including *S*. Typhi, that carry *zot* gene are very limited (positive BLASTP hit only to *Salmonella enterica* subsp. salamae and *Salmonella enterica* subsp. houtenae in NCBI nr database as of 10 May 2014) in our phylogenetic analysis. The *S*. Typhi *zot* homologue formed a monophyletic group with various *zot* homologues from Enterobacteriacea, including *E. coli* and *Yersinia pestis* but grouped more distantly with the well characterised Zot protein in *Vibrio cholerae* and *Neisseria meningitidis*[47] (Figure 4). Zot was previously characterised as uropathogenic-specific protein in uropathogenic *E. coli* and a potentially important toxin in many pathogens that have received minimal attention [48]. Recently, *Campylobacter concisus*, isolated from the patients with inflammatory bowel disease, was the only species among the Campylobacteriales to harbour zot, suggesting its importance in gastrointestinal pathogenesis [49]. However, little is understood on the mechanism of action of this protein, although earlier study of Zot in *V. cholera* suggested that the protein act as a toxin that disrupts the integrity of the intestinal barrier by targeting the tight junction to increase tissue permeability [50, 51].

Our phylogenetic tree analysis (Figure 4) has further revealed that the *zot* homologue is surprisingly diverse, being present in the genomes of plant pathogens, such as *Ralstonia solanacearum* and *Xanthomonas campestris*, and opportunistic pathogens that are found in soil and water, such as *Providencia stuartii* and *Pandoraea* spp. Interestingly, this *zot* homologue was also found in lithoautotrophic bacteria, such as *Caminibacter mediatlanticus*, *Sulphurimonas* spp. that clustered closely with the functionally validated Zot in *Neisseria* spp. and *Campylobacter* spp. but more distantly related to Enterobacteriacea, including our *S*. Typhi. Other species such as *Pseudogulbenkiana ferroo*xidans and *Beggiatoa alba*, which were isolated from extreme environments, such as deep hydrothermal vents and sulphur springs were found to be more closely related and shared a common ancestry with *S*. Typhi (Figure 4). The importance of the necessity of gene targeting tight junctions in these extremophiles is yet to be understood but may be due to the role of the toxins in facilitating the penetration of the tissue layers that cover the host organisms that they colonise.

Despite harboring four highly conserved domains leading to its putative assignment as a Zot toxin, the *S*. Typhi Zot lacks a previously identified active domain (FCIGRL) found in *V. cholera*. Nevertheless, a previous study has shown that the partial refolding of the denatured binding peptide of this domain did not prevent its specific binding to the Zot receptor on Caco-2 cells, demonstrating that the conformationally varied domain was still able to induce toxin activity [52]. The possible mechanism of Zot in S. Typhi may be similar to that of *V. cholerae*, in which tight junction disassembly is induced through the activation of the proteinase-activated receptor 2 [53]. However, comparison of the predicted Zot protein tertiary structures of S. Typhi with that of *V. cholerae* and *N. meningitidis* showed that they were highly variable, suggesting that the toxin might have different mechanisms of action despite maintaining some core binding activities.

The high similarities between the GIs of the BL196 and CR0044 strains further provide strong evidence of the common ancestry between the strains. Alarmingly, these strains are more diverse than was previously thought, emphasising our concern that such strains that have acquired new genes through HGT are circulating in the country or elsewhere.

### Microvariations distinguishing closely related strains

Single nucleotide polymorphisms (SNPs) were determined for the comparisons of the two highly related strains, BL196 and CR0044. We investigated the microvariations of the high quality non-synonymous single nucleotide polymorphism (nsSNPs) that were identified. In this study, only the potential functions altering the nsSNP mutations were considered. Despite being highly related and similar, the genomes could be discretely distinguished from each other by 29 nsSNPs (Additional file 6). Interestingly, the 29 nsSNPs that were identified, two were found on two highly related virulence determinant genes, RNA polymerase sigma factor *rpoS* and the Vi polysaccharide biosynthesis protein *tviE*. These two mutations are strain-specific and were not detected in any other *S*. Typhi genomes that were analysed. BL196 carried a mutant *rpoS* (P193L), which is 100% similar to *Salmonella* Pullorum S6702, the causative agent of fowl typhoid [54]. Interestingly, unusual *rpoS* gene was also observed in P-stx-12. The *rpoS* of P-stx-12 contains an additional 57 bp of a response regulator *gacA* fragment that was fused at the 5’ end of the gene, resulting in a longer *rpoS*. The *gacA* fragment was highly similar to the transposons *Tn10d tetA* and *tetR*, which are associated with regulatory and transcription signals, suggesting the occurrence of transposition events and possibly gene regulation. The *rpoS* mutant and its effects on *S*. Typhi were first reported in the Ty2 genome and attenuated strain Ty21a. These mutants, which were partially derived from natural mutations in Ty2, were found to affect the stress response and other related functions significantly [21, 55]. Mutations in *rpoS* are apparently advantageous to the strain for survival in the host during prolonged stress, allowing for the selection of more efficient transcription factors for survival and fitness during unfavourable conditions [56]. *rpoS* is commonly associated with the virulence regulation of pathogens because it regulates over 30 genes that are related to the stress response, such as the Spv protein, which is involved in host cell survival, and Vi-polysaccharide biosynthesis proteins in different osmolarities [57].

In contrast, CR0044 carried a mutant TviE (H53Y). TviE, which is encoded by SPI-7, is required for virulence capsular formation and acts as a protective antigen. The antibody that responds to the Vi-positive strain has been shown to be more virulent than that targeting the Vi-negative strain [58, 59]. A role of the RpoS protein in fine-tuning the synthesis of the Vi polysaccharide in *S*. Typhi has also been reported [60], suggesting the possible close regulation of these two genes in modulating adaptation and virulence capabilities in different host environments. The nsSNPs that were detected in these two closely related genes may indicate that their adaptive selection is important for host survival. To address false-positive results, the nsSNPs were validated using a high-resolution melt (HRM) analysis and direct sequencing (Additional file 7). The unique HRM profiles of the wild-type strains and those containing the SNP transition mutations in the normalised graph are shown in (Additional file 7a and b). Both SNPs showed unique melting profiles for the strains that were tested. For the *rpoS* SNP, the transition mutation from C to T occurred in strain BL196, and the separation of the melting profile began at ~81°C and ended at 84°C. For the *tviE* SNP, the transition mutation from C to T occurred in strain CR0044, and the separation of the melting profile began at ~74°C and ended at 78°C. The results of this analysis were further confirmed by the direct sequencing of targeted loci (Additional file 7a and b). The primers and HRM profiles that were developed may be useful for distinguishing between these two strains and as important markers for future surveillance. Another 27 high quality nsSNPs were also identified that mainly encoded for non-virulence factors (Additional file 6). Twenty-three genes had well defined functions, including four that were involved in metabolism and 12 that played roles in cellular processes, signalling and transport. The remainder were poorly characterised or had unknown functions. Additionally, out of 29 nsSNPs, 24 (83%) mutants were detected in the carrier strain CR0044, suggesting the possible functional adaptation of this carrier strain in the host cell relative to its closely related strain.

However, most of the mutant SNPs that were carried by CR0044 was not detected in P-stx-12 with the exception of the gene encoding the trehalose permease IIC component. In fact, these two carrier strains are genetically different, with the presence of 253 SNPs, including 159 nsSNP. Notably, the nsSNPs were mainly found in the genes encoding proteins associated with virulence, metabolism, outer membrane, and other regulatory proteins. However, nsSNPs were also observed in a large number of uncharacterised proteins, suggesting that independent genomic factors may have contributed to the carrier state.

It is important to note that the independent mutations in *rpoS* and *tviE* as well as other genes in strains BL196 and CR0044 support the postulation that these strains possibly diverged from a common ancestor. We speculate that the source of this ancestral strain may be still circulating in the country. Therefore, national surveillance program and epidemiologic study are pivotal for effective microbial source tracking and dissemination control.

### Analysing the molecular effects of nsSNPs, protein structure modelling and molecular dynamics (MD) simulation

Point mutations that cause alterations in amino acids can have profound effects on the structural stability of proteins; hence, a study of their effects is necessary to understand their functionalities. We have modelled both the native and mutant structures of the proteins. Out of the 5 native protein structure models that were generated by I-Tasser [61], the best structure with the highest confidence score (C-scores: RpoS, 0.64; TviE, −0.40) was collected and used for further investigations. The modelled native RpoS and mutant RpoS structures showed good stereochemical properties, with 92.0% and 85.4% of the residues being within the most favourable region of the Ramachandran plot, respectively, whereas the native and mutant TviE showed 86.5% and 80.0% of the residues in most favourable region of the plot, respectively. All of the structures passed ProSa model quality validation with Z-scores (RpoS native, −5.61; RpoS mutant, −5.57; TviE native, −6.32; TviE mutant, −6.56) falling well within the range of those that are typically reported for native proteins of similar sizes from different sources (X-ray, NMR).

**Figure 5:**
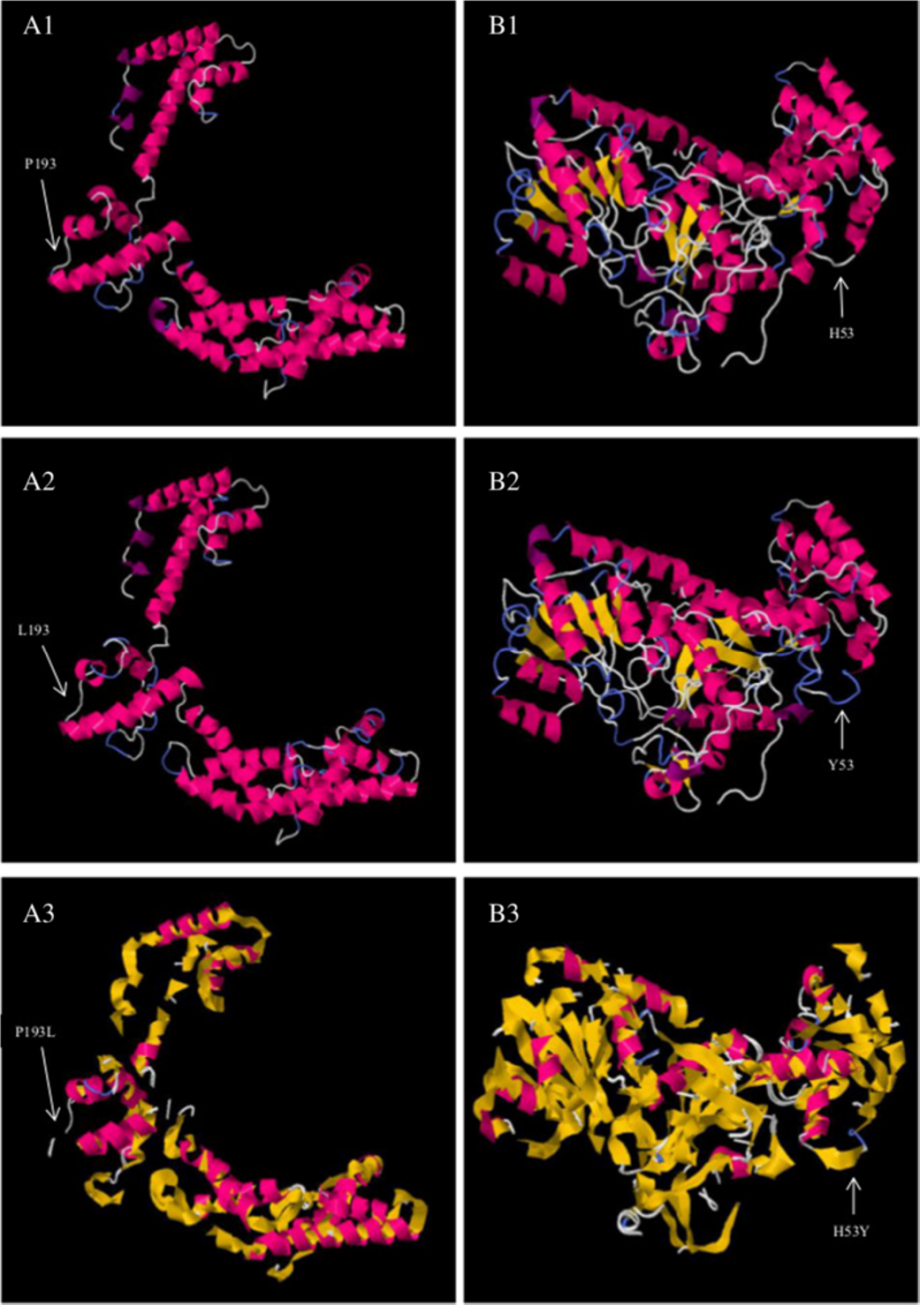
**Modelled protein structures of RpoS and TviE.** A1 showed native RpoS with proline at position 193. A2 showed mutant RpoS with amino acid leucine at position 193. A3 showed superimposed structure of RpoS native structure (yellow) with mutant structure (pink). B1 showed native TviE with histidine at position 53. B2 showed mutant TviE with amino acid tyrosine at position 53. B3 showed superimposed structure of TviE native structure (yellow) with mutant structure (pink).

Molecular dynamics (MD) simulation with a realistic aqueous solvent environment was performed to reveal the explicit solvent behaviours of the native and mutant structures, which could elicit the differences in their dynamics and stabilities. The energy minimisation studies were performed for both the native and mutant structures, and the total energies of the native and mutant RpoS achieved were −4115.46 J/mol and −4804.66 J/mol, respectively, whereas those of the native and mutant TviE achieved were −3203.54 J/mol and −2906.31 J/mol, respectively. Energy minimisation assessments provide clues regarding protein stability. The deviation between two structures can be evaluated by the root mean square deviation (RMSD). The higher the RMSD value is, the greater the deviation will be between the native and mutant structures. The RMSD value between the native and mutant RpoS was found to be 0.39 Å and that between the native and mutant TviE was 0.43 Å. The native, mutant, superimposed protein structures at their corresponding positions for RpoS and TviE are shown (Figure 5). The RMSD values of the native and mutant structures were significantly similar for both RpoS and TviE, suggesting similar levels of protein folding alterations. We analysed the molecular effects and functional modifications based on several predictive tools (refer to Methods) targeting various aspects of protein dynamics with confidence scores (Additional file 8). Out of 9 predictive tools used, all found that the nsSNP in *rpoS* was deleterious (affecting protein structure and function), whereas in t*viE*, 3 predictive tools found that the nsSNP to be deleterious, 5 to be neutral and one undetermined. The nsSNP in *rpoS* showed PSIC score of 1.0 (deleterious) with PolyPhen2 [62] in addition to a probability score of 0 (<0.05, deleterious) with SIFT [63] and a Provean [64] score of −9.261 (<−2.5, deleterious), suggesting that the nsSNP could affect the protein drastically and result in functional modifications. It is important to note that the deleterious effects could lead to positive or negative functional modifications. To further validate the results, a method that was based on the hidden Markov model (HMM) from PANTHER [65] was used. The nsSNP in *rpoS* was found to be deleterious, but undetermined for *tviE*.

The predictions of the nsSNP in *rpoS* by these tools are in agreement and show strong correlations among various methodologies. The analysis with I-Mutant 3.0 [66], which is based on the support vector machine (SVM) and DGG stability changes, revealed that the nsSNP in *rpoS* may have led to its greatly increased stability (DGG value of 0.89 Kcal/mol, >0.5 kcal/mol indicates large increase in protein stability), suggesting that the deleterious effects of the nsSNP is favourable to the protein folding and structure and possibly lead to enhanced functions. The DDG value is calculated from the unfolding Gibbs free energy value of the mutated protein minus the unfolding Gibbs free energy value of the wild-type strain (Kcal/mol), which is based on a trained cross-validation procedure using a comprehensive experimental database of protein mutations [66]. These predicted results were consistent with the differences in the energy minimisation of MD simulation, suggesting the favourable folding of the mutant structure. Native and mutant structures differ due to the specific properties of the residues that could disrupt the structure and function of the protein. The mutant residue (leucine) of RpoS is molecularly larger in size than the wild-type residue (proline), which is a highly rigid, small molecule that is required to induce a unique backbone conformation. However, the alteration from the small-sized secondary amine structure to a larger-sized primary aliphatic amine at this site can disturb its conformation and lead to bumps in the structure, in which the mutant residue is not in the correct position to make the typical hydrogen bond that is formed by the native residue (Figure 5). The hydrophobicity of the wild type and mutant differ because the mutation introduces a very high hydrophobic residue in place of a less hydrophobic residue. This can result in a loss of hydrogen bonding and may disturb proper protein folding. The wild-type proline residue in RpoS is well conserved and is located at the discrete compact three-helical domain within region 3 of the protein; however, no known mutant residues with similar properties were observed at this position in the other homologous sequences. This region is the specific binding site of bacterial promoters containing an extended −10-promoter element and is primarily involved in the binding of the core RNA polymerase in the holoenzyme. The mutation in this important site could affect protein functioning pertaining to the transcription efficiency.

However, the predictions regarding the nsSNP in *tviE* were contrasting, which suggest more benign effects, indicating that nsSNPs may have more modest effects on the functioning of this protein. The nsSNP in *tviE* may have decreased its stability (DGG value of −0.54 Kcal/mol, with < −0.5 kcal/mol indicating large decrease in protein stability) as predicted by I-Mutant 3.0 [66]. Unlike RpoS, the mutant residue (tyrosine) in TviE is not conserved at this position of the helical structure, and other non-similar residues were observed at this position with no known protein binding sites in other homologous sequences. However, the size of the mutant residue (tyrosine) is larger compared with that of the native residue (histidine) despite the fact that both are polar and located at the surface of the protein. This mutation also introduces a more hydrophobic residue at this position, suggesting that it could possibly alter the correct folding of the protein, subsequently affecting hydrogen bonding but with only modest effects. These novel mutations could have important impacts on the pathogenesis and persistence of strains in the host. Although we could not determine the true extent of the effects of the nsSNPs on the protein functions, these data suggest that they alter the protein structures (and possibly their functions) considerably, potentially leading to the enhanced regulation of RpoS and stress response in BL196 and reduced efficiency of TviE in the virulence capsular formation of CR0044. The close regulation of these two genes may be relevant to the virulence and persistence capabilities of the closely related strains that lead to the different clinical outcomes. These data provide essential insights into the underlying molecular mechanisms upon mutations and serve as caveats for future functional gene knock-out studies.

## Conclusions

We have thoroughly dissected the genome of *S*. Typhi in association with three important epidemiological settings. Comparative genomics and phylogeny analyses have revealed that the strain that was associated with the large outbreak was highly related and shared common ancestry with the carrier strain. These findings are supported by their common genomic features and uncommon gene repertoires, including dispensable genes, phages and an additional putative pathogenomic island harbouring virulence-related genes, and *zot* in particular*.* Apart from these, variations were also identified in T6SS and SPI-related genes, insertion sequences, CRISPRs and nsSNPs among the studied genomes, which may be novel factors that contribute to the varied host adaptations and pathogenicities. Despite being highly similar, BL196 and CR0044 may be distinguished by microvariations in their nsSNPs. Interestingly, the protein modelling and MD simulation of the wild-type and mutant RpoS and TviE suggest that the potential protein structure and functional modification was more stable in RpoS, which plausibly leads to enhanced regulation and stress response. On the other hand, the mutation in TviE was less stable than that of the wild type, which could potentially lead to lower capsular formation efficiency. The close association of these virulence-related genes are relevant for long-term host persistence and adaptation, which serve as important caveats for further functional studies. The analysis also revealed that SPI-10, which was previously thought to be relatively stable, is possibly prone to excision. Moreover, multiple regions of genomic plasticity were detected. In particular, the discovery of new GIs in the outbreak strain and the highly related carrier strain are of great concern epidemiologically. These results suggest the plasticity and open pan-genome of *S*. Typhi, indicating that the pathogen is more diverse than previously thought and that genes may have been acquired or transferred from one another through HGT, posing higher risk for effective disease control. The genomic information that was obtained in this study provides novel insights into the pathogenesis and control of *S*. Typhi, essentially, gene targets for vaccine development.

## Methods

### Choice of strains

Three Malaysian *S*. Typhi strains (BL196, ST0208 and CR0044) were selected for the comparative genomic analysis that were based on previous PFGE data and reported genome sequences [6, 17, 18]. These strains are associated with diverse epidemiological settings. Strain BL196 was isolated from a typhoid patient with diarrhoea during a large outbreak in Kelantan, Malaysia that resulted in 735 cases and 2 deaths in the year 2005. Strain ST0208 was isolated from a typhoid patient, who was a sporadic case, at a local tertiary hospital in Kuala Lumpur, Malaysia. Strain CR0044 was isolated in 2007 from a carrier (food handler) following the large 2005 outbreak in Kelantan, Malaysia (Table 1). The initial molecular analysis showed that both the CR0044 and BL196 strains were highly similar with only one band difference as revealed by PFGE (Additional file 2). These 3 new genomes were compared with all three of the available *S*. Typhi full genomes at the time of our analysis (CT18, Ty2 and P-stx-12). We compared our strains with CT18 and Ty2, the former is a fairly recent and geographically related multidrug-resistant strain that was isolated from Vietnam, the latter was isolated from Russia in the early 1970s, a geographically more distant strain known to be used for oral typhoid vaccine development. We also performed a comparison using a carrier associated strain, P-stx-12, which was isolated from a carrier in India. All of these strains represent *S*. Typhi from diverse temporal and spatial backgrounds in association with variable epidemiological settings. The details of the bacterial strains are provided in Table 1 [GenBank accession number: BL196 (AJGK00000000.1), ST0208 (AJXA00000000.1), CR0044 (AKZO00000000.1), CT18 (AL513382), Ty2 (AE014613) and P-stx-12 (CP003278).

**Table 1:** **Details of bacterial strains used in this study**

Strain name ^a^	Year of isolation	Location of isolation	Specimen ^b^	Epidemiological information ^b^ (if available)
BL196	2005	Kelantan, Malaysia	Blood	Outbreak
CR0044	2007	Kelantan, Malaysia	Stool	Carrier
ST0208	2008	Kuala Lumpur, Malaysia	Stool	Sporadic
CT18	1993	Mekong Delta, Vietnam	Blood	NA
Ty2	1916	Russia	NA	NA
P-stx-12	2009	Varanassi, India	Stool	Carrier

^a^ *S*. Typhi strains and their Genbank accession numbers: BL196 (AJGK00000000.1) [17], CR0044 (AKZO00000000.1) [18], ST0208 (AJXA00000000.1) [6], CT18 (AL513382) [20], Ty2 (AE014613) [21] and P-stx-12 (CP003278) [22]. ^b^ *NA*: Not available.

### DNA sequencing, assembly and annotation

Previous sequencing was carried out on 3 *S*. Typhi strains using the Illumina Genome Analyser (GA2X, pipeline version 1.6, insert size 300), generating >10 total gigabytes of data. A *de novo* assembly and annotations were carried out and further validated with various pipelines as previously described [6, 17, 18].

### Multilocus sequence typing

Multilocus sequence typing (MLST) housekeeping gene sequences (*thrA* (aspartokinase + homoserine dehydrogenase), *purE* (phosphoribosylaminoimidazole carboxylase), *sucA* (alpha ketoglutarate dehydrogenase), *hisD* (histidinol dehydrogenase), *aro*C (chorismate synthase), *hemD* (uroporphyrinogen III cosynthase) and *dnaN* (DNA polymerase III beta subunit) according to PubMLST were extracted from the genome sequences [19]. The alignments for each of these genomic regions were bioinformatically extracted, trimmed and concatenated into final sequence lengths of 3,336 bp using MEGA 5 [67]. The sequences were subsequently submitted to the MLST database (http://mlst.warwick.ac.uk) and assigned existing or novel allele type numbers. The composite sequence types (STs) were defined by the database based on the allelic profile that was derived from each of the seven loci. The STs from the fragmented incomplete genomes were derived by comparing the 3 less conserved alleles *hemD*, *his*D and *thrA,* while assuming that the other 4 alleles, *aroC*, *dnaN*, *purE* and *sucA,* were conserved. The results with positive BLASTN hits of 100% query sequence coverage (E < 1 × 10^−6^) were only considered in the analysis.

### Comparative genomic analysis

Protein coding gene predictions were performed using Prodigal [68]. The predicted genes were then subjected to annotations using Blast2GO [69] (E < 1 × 10^−30^). The genomic sequences and functional annotations of the CDSs were validated based on the results of homology searches against the public non-redundant nucleotide and protein databases (http://www.ncbi.nlm.nih.gov/) using BLASTN and BLASTP [70] The genes were selected based on the top BLAST hits (E < 1 × 10^−30^, ≥60% query coverage and ≥60% protein identity). The open reading frames (ORFs) of the genomes were reciprocally compared (ORF-dependent comparisons) using RAST [71]. The subsystem category distributions were compared among the genomes. The circular map of genes that was based on the similarities of the amino acid sequences of the BL196, CR0044, ST0208, TY2 and P-stx-12 genomes against that of CT18 was generated using the BLAST Ring Image Generator (BRIG) [72]. A synteny-based analysis was performed by aligning the genomes with CT18 as a reference, and the contigs were reordered with iterative refinements using progressiveMauve [73] and Nucmer [74]. The best alignments were chosen for the multiple genome alignments. The reference-ordered and -oriented genomic scaffold that was used for the subsequent analysis was generated by concatenating reordered contigs by inserting 5Ns between the contigs using an in-house python script. A bioinformatic pipeline using the Pan-Genomes Analysis Pipeline (PGAP) [75] was utilised to identify the homologous regions of the compared ORFs at an E value cut-off of 1 × 10^−10^. Then, the nucleotide and amino acid sequences of the query ORF and selected target homologous regions were aligned and validated using BLAST against the NCBI redundant database. The resulting matched and validated homologues, paralogues and orthologues were used for the multiple alignment comparison. A genomic island analysis and prediction were performed using IslandViewer [43], which includes 3 methods (Island Pick, IslandPath-DIMOB and SGI-HMM). The IS elements were analysed by IS Finder (http://www-is.biotoul.fr). The phages were analysed using PHAST [76]. The regions that were algorithmically identified as intact and those sharing high similarities were compared and analysed. The sequence content comparison was performed using ACT [77] and MEGA 5 [67]. The regions of interest were then manually curated to improve the annotations and gene predictions. The nsSNP analysis was carried out using PGAP [75] by sorting them from synonymous SNPs, deletions and insertions, and the results were validated using the CLC Genomic Workbench version 5.1 (CLC Bio, Aarhus, Denmark).

### Phylogenomic analysis

The genome sequences of 20 global [*S*. Typhi strains and their Genbank accession numbers were as follows: BL196 (AJGK00000000.1), CR0044 (AKZO00000000.1), ST0208 (AJXA00000000.1), UJ308A (AJTD00000000.1), UJ816A (AJTE00000000.1), CR0063 (AKIC00000000.1), 404ty (CAAQ00000000.1), E00-7866 (CAAR00000000.1), E01-6750 (CAAS00000000.1), E02-1180 (CAAT00000000.1), E98-0664 (CAAU00000000.1), E98-2068 (CAAV00000000.1), J185 (CAAW00000000.1), M223 (CAAX00000000.1), AG3 (CAAY00000000.1), E98-3139 (CAAZ00000000.1), STH2370 (JABZ00000000.1), CT18 (AL513382), Ty2 (AE014613) and P-stx-12 (CP003278) (Additional file 9). These sequences were submitted to the Reference Sequence Alignment-based Phylogeny Builder (RealPhy) [78] for the identification of sites that were relevant for the phylogenomic analysis using the default parameters. The complete genome of *S*. typhi CT18 was chosen as the reference genome, and all of the query genomic sequences were divided into possible sequences of 50 bp (default) and subsequently mapped to the reference genome via Bowtie2 with a default k-mer length of 22, allowing for one mismatch within the k-mers to maximise sensitivity. The generated multiple genome sequence alignments were subsequently used to construct an unrooted phylogenetic tree that was inferred via the approximate maximum likelihood method using FastTreeMP [79].

### Phylogenetic analysis of *zot*

The *zot* amino acid sequence data from 40 closely and distantly related bacterial strains were downloaded from the Genbank. The sequences from both BL196 and CR0044 were aligned with those of the 40 bacterial strains that were selected using the MAFFT [80] E-INS-I strategy. A phylogenetic analysis was subsequently performed using maximum likelihood phylogenetic algorithms with the PhyML module of SeaView V4.5 [81], which was supported by 1000 bootstrap replicates. The Maximum Likelihood tree was constructed using the best substitution model (Blosum62 algorithm) after being tested and optimised by ProtTest 2.4 [82].

### PCR validation of selected genes and SNPs

PCR was carried out to validate the identified high-quality nsSNPs. Genomic DNA for the sequencing reactions was extracted using the Wizard® Genomic DNA Purification Kit (Promega, Madison, WI, USA). The amplification of the selected genes was performed using a standard PCR protocol. Each 25 μl PCR reaction contained 150 μM (each) deoxynucleoside triphosphates, 1× PCR colourless buffer, 1.2 mM MgCl_2_, 0.2 μM of primer and 0.5 U of Go Taq Flexi DNA Polymerase (Promega, Madison, WI, USA). The PCR was performed under the following conditions: initial denaturation at 95°C for 30 s, 30 cycles of denaturation at 95°C for 30 s, 30 s at the respective annealing temperature (Additional file 10) and an extension step at 72°C for 40 s; a final extension was performed at 72°C at 1 min. The reactions were carried out using a PCR Master Cycler (Eppendorf AG, Hamburg, Germany). The primer sets that were used for the target genes are shown (Additional file 10).

### Sequencing and high-resolution melting (HRM) analysis

The PCR products were purified using the PCR Clean-up Kit (Qiagen, Valencia, CA) according to the manufacturer’s instructions. The PCR products were then sent to a commercial sequencing facility (First BASE Laboratory Sdn Bhd, Selangor, Malaysia) for direct sequencing. The nsSNP variations were validated with a pair of primers as described (Additional file 10) and subsequently used for a high-resolution melting (HRM) analysis using the Kapa HRM Fast PCR Kit (Kapa Biosystems, Boston, Massachusetts, USA) and Eco Real-Time qPCR System (Illumina, San Diego, California, USA) according to the manufacturer’s instructions. The melting curve profiles that were generated were analysed with the Eco-qPCR software using both homozygous and heterozygous controls.

### Analysis of molecular effects of nsSNPs and protein structure modelling

The nsSNPs of *tviE* and *rpoS* were selected for the predictions and analyses of the further molecular effects. The Poly-Phen2 [62], SIFT [63], Provean [64], SNAP [83], I-Mutant 3.0 [66] and PredictSNP 1.0 (PredictSNP, MAPP, PhD-SNP and Panther) [84] tools were used to examine the functional modifications and predictions of the tolerated and deleterious nsSNPs. The details of the methods and scores that were used for each tool are included in (Additional file 8). A combination of different prediction methods were used to increase the prediction accuracy and confidence. The protein structures of TviE and RpoS were modelled using the I-TASSER server [61]. The best model was selected based on the optimal C-score. Further, the native structure was mutated by introducing a point mutation in the native RpoS protein at P193L (proline to leucine) and native TviE protein at H53Y (histidine to tyrosine) using FixPDB with the NOMAD-Ref server [85] and validated with the SPDB viewer [86]. The native and mutant structures were checked, fixed, refined and energetically optimised by MDWeb [87], ModRefiner [88], FG-MD [89] and the SPDB viewer [86]. The qualities of the model structures were independently verified with the PROCHECK [90], WHATCHECK [91] and PROSA programs [92].

### Molecular dynamics (MD) simulation and energy minimisation

The molecular dynamics (MD) simulations were carried out using MDWeb [87]. The optimised structures of the native and mutant RpoS and TviE proteins were used as input data for the MD simulations. GROMACS topologies were first generated by removing the crystallographic water molecules and adding missing side chain and hydrogen atoms. Histidine residues were protonated according to the protpKa program algorithm with the GROMACS package. Water molecules were added at energetically favourable positions of the structure surfaces. Hydrogen atoms were energetically minimised for 500 steps of hydrogen conjugate gradients, while the remainder of the structures were fixed and followed by energy minimisations for 500 steps of conjugate gradients, restraining heavy atoms with a force constant of 500 KJ/mol.nm^2^ to their initial positions. The system was solvated with simple point charge (SPC) water molecules at spacing distances of 15 Å around the molecules. Chloride (Cl-) and/or sodium (Na+) ions were added until the system was neutralised at a concentration of 50 mM. The step involving the minimisations of the structures for 500 steps of conjugate gradients to restrain the heavy atoms with a force constant of 500 KJ/mol.nm^2^ to their initial positions was repeated. The whole molecular system was subjected to energy minimisations of 500 iterations by a steepest descent algorithm implementing a GROMOS96 43a1 force field. The comparative analysis of structural deviations between the native and mutant proteins of RpoS and TviE was assessed by their respective RMSD values.

## Electronic supplementary material

Additional file 1: **Genomic features of** ***S*** **. Typhi strains.** (XLSX 10 KB)

Additional file 2: **Pulsed-field gel electrophoresis of** ***S*** **. Typhi strains BL196, CR0044 and ST0208.** (PDF 220 KB)

Additional file 3: **Strains used to detect prevalence of** ***zot*** **in** ***S*** **. Typhi using PCR.** (XLSX 12 KB)

Additional file 4: **Representative gel picture of** ***zot*** **prevalence in** ***S*** **. Typhi.** (PDF 272 KB)

Additional file 5: **Primers used for** ***zot*** **prevalence screening in** ***S*** **. Typhi strains.** (XLSX 10 KB)

Additional file 6: **nsSNPs detected in** ***S*** **. Typhi strains BL196 and CR0044.** (XLSX 11 KB)

Additional file 7: **a: High-resolution melting profile of** ***rpoS*** **fragment in normalised graph mode.** b: High-resolution melting profile of Vi-polysaccharide biosynthesis *tviE* fragment in normalised graph mode. (ZIP 357 KB)

Additional file 8: **Prediction of nsSNP effects on rpoS and tviE using multi-predictive tools.** (XLSX 11 KB)

Additional file 9: **Strain backgrounds used in phylogenomic analysis.** (XLSX 12 KB)

Additional file 10: **Primers used for PCR, direct sequencing and high-resolution melt analysis for nsSNP detected in** ***S*** **. Typhi strains BL196 and CR0044.** (XLSX 9 KB)
