# Supplementary material for: Comparative genomics of closely related Salmonella enterica serovar Typhi strains reveals genome dynamics and the acquisition of novel pathogenic elements
Source: BMC Genomics. 2014 Nov 20;15(1):1007. doi: 10.1186/1471-2164-15-1007 (PMC4289253; doi:10.1186/1471-2164-15-1007)
Supplement: Supplementary file 2 — Additional file 2: Pulsed-field gel electrophoresis of S . Typhi strains BL196, CR0044 and ST0208. (PDF 220 KB) [file 12864_2013_6828_MOESM2_ESM.pdf]

Pulsed-Field Gel Electrophoresis of *S*. Typhi Strains (BL196, CR0044, ST0208)

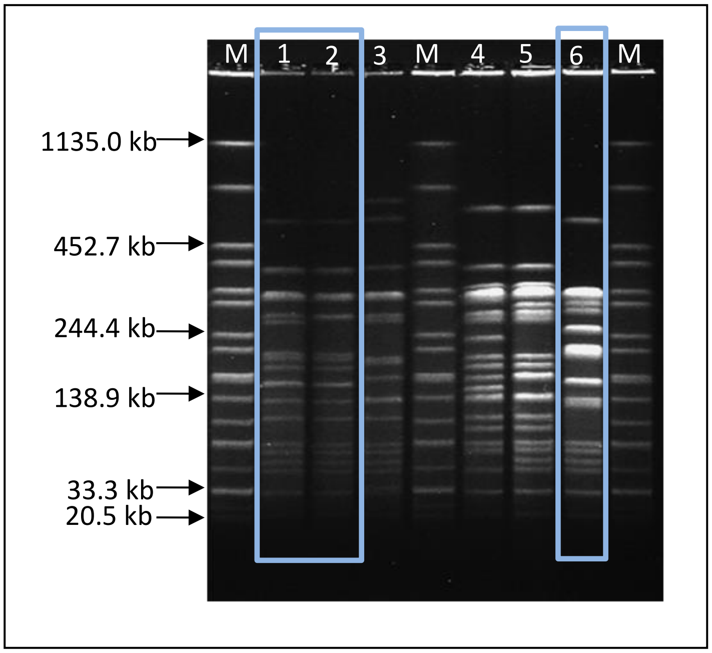

Additional file 2. Pulsed-Field Gel Glectrophoresis (PFGE) pattern of Salmonella Typhi following digestion with *Xba*1. M = *Salmonella* Braenderup H9812 markers; Lane 1= BL196; Lane 2= CR0044; Lane 3= CR0063; Lane 4= UJ308A; Lane 5= UJ816A; Lane 6= ST0208. Values on the left (in kilobase pairs) refers to the position of marker bands.
