# Supplementary material for: Comparative genomics of closely related Salmonella enterica serovar Typhi strains reveals genome dynamics and the acquisition of novel pathogenic elements
Source: BMC Genomics. 2014 Nov 20;15(1):1007. doi: 10.1186/1471-2164-15-1007 (PMC4289253; doi:10.1186/1471-2164-15-1007)
Supplement: Supplementary file 4 — Additional file 4: Representative gel picture of zot prevalence in S . Typhi. (PDF 272 KB) [file 12864_2013_6828_MOESM4_ESM.pdf]

Additional file 4: The representative gel picture of *zot* prevalence in *S*. Typhi.

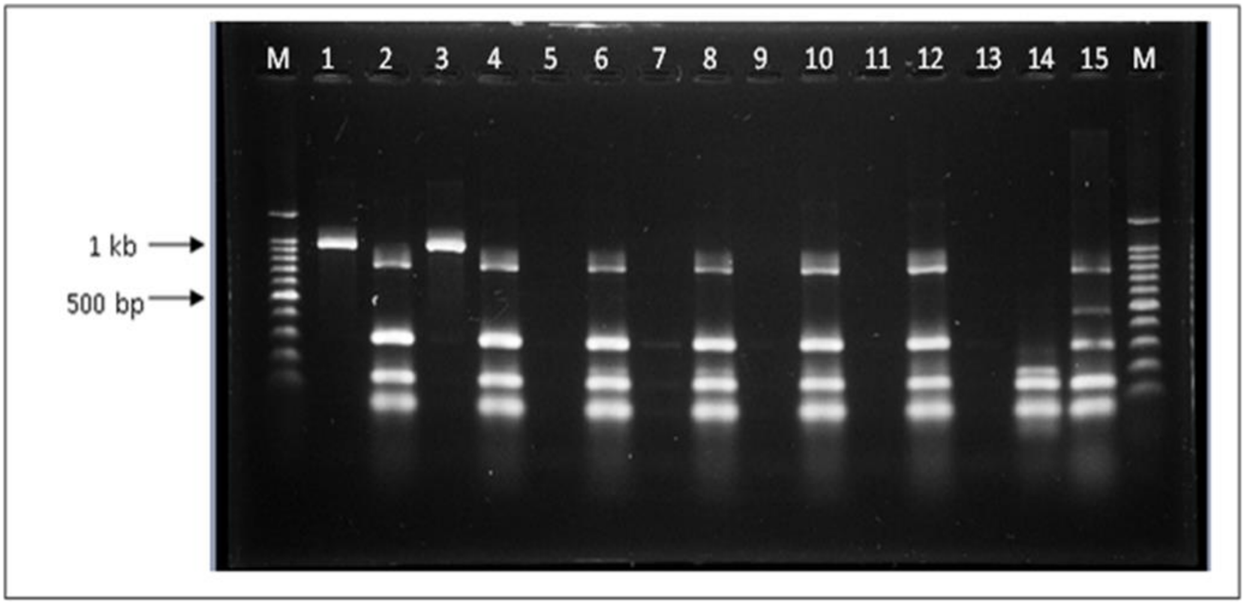

Additional file 4: The representative 1.5 % gel electrophoresis picture of *zot* prevalence screening using *zot* PCR detection coupled with in-house *S.* Typhi detection multiplex PCR. Lane M= 100 bp Markers, Lane 1= *zot* presence for BL196; Lane 2 = BL196; Lane 3= *zot* detected for CR0044 ; Lane 4= CR0044; Lane 5= *zot* was not detected for ST0208; Lane 6= ST0208; Lane 7, 9, 11= *zot* was not detected for other *S*. Typhi strains tested; Lane 8, 10, 12= other *S*. Typhi strains tested; Lane 13= Negative control for *zot*; Lane 14= negative control for in-house *S.* Typhi detection multiplex PCR; Lane 15 = Positive control (187 bp: internal amplification control; 332 bp: *S*. Typhi; 496 bp: *S*. Paratyphi A; 784: *Salmonella enterica*). Amplicon size of 1026 bp correspond to *zo*t detected in BL196 and CR0044.
