# Supplementary material for: Comparative genomics of closely related Salmonella enterica serovar Typhi strains reveals genome dynamics and the acquisition of novel pathogenic elements
Source: BMC Genomics. 2014 Nov 20;15(1):1007. doi: 10.1186/1471-2164-15-1007 (PMC4289253; doi:10.1186/1471-2164-15-1007)
Supplement: Supplementary file 7 — Additional file 7: a: High-resolution melting profile of rpoS fragment in normalised graph mode. b: High-resolution melting profile of Vi-polysaccharide biosynthesis tviE fragment in normalised graph mode. (ZIP 357 KB) [file 12864_2013_6828_MOESM7_ESM.zip › 1724553800106302_add8a.pdf]

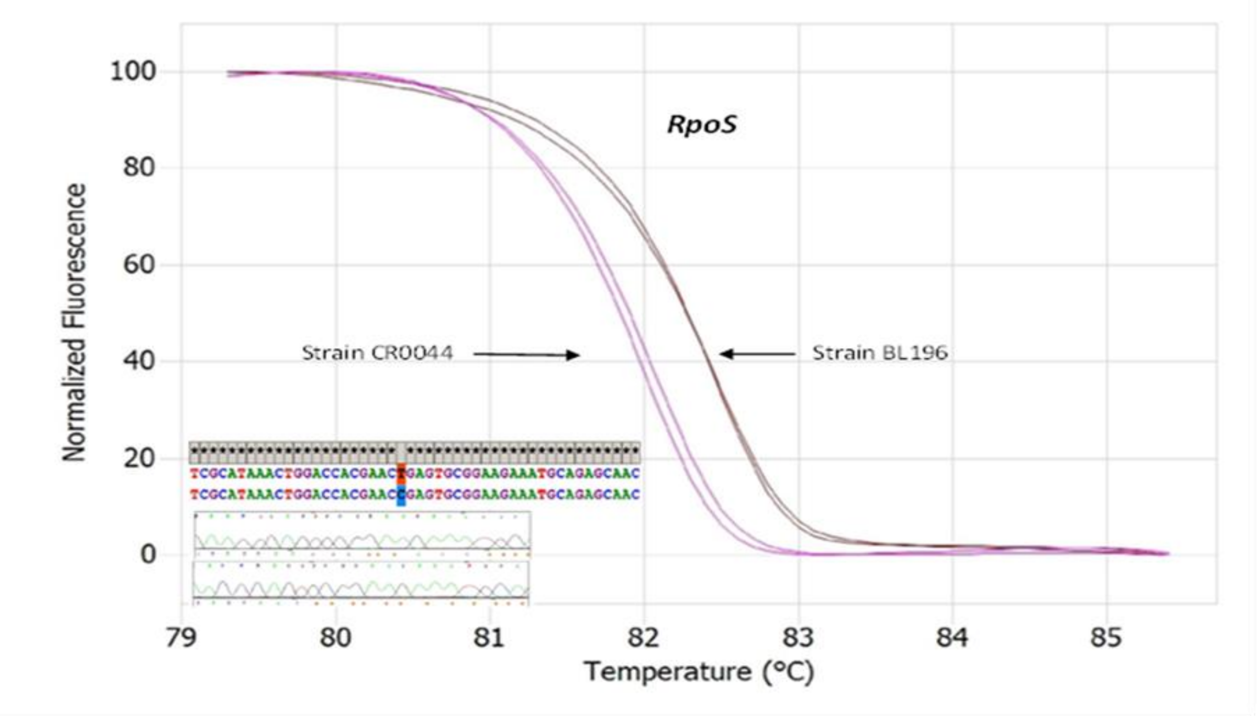

Additional file 9a: High resolution melting profile of rpoS fragment in the normalized graph mode. The normalized fluoresence (y-axis) is plotted against temperature in °C (x-axis). The pink colour curve denodes strains CR0044 and brown denodes BL196 as labeled. Deviation of pattern can be clearly observed. The SNP are validated with Sanger sequencing as shown on the chromatogram. The SNP region of rpoS is aligned between BL196 and CR0044 using MEGA 5 is shown.
